# Forecasting the Post-Pandemic Effects of the SARS-CoV-2 Virus Using the Bullwhip Phenomenon Alongside Use of Nanosensors for Disease Containment and Cure

**DOI:** 10.3390/ma15145078

**Published:** 2022-07-21

**Authors:** Mohammed S. Alqahtani, Mohamed Abbas, Mohammed Abdulmuqeet, Abdullah S. Alqahtani, Mohammad Y. Alshahrani, Abdullah Alsabaani, Murugan Ramalingam

**Affiliations:** 1 Radiological Sciences Department, College of Applied Medical Sciences, King Khalid University, Abha 61421, Saudi Arabia; mosalqhtani@kku.edu.sa; 2 BioImaging Unit, Space Research Centre, Department of Physics and Astronomy, University of Leicester, Leicester LE1 7RH, UK; 3 Electrical Engineering Department, College of Engineering, King Khalid University, Abha 61421, Saudi Arabia; mabdulmuqeet@kku.edu.sa; 4 Computers and Communications Department, College of Engineering, Delta University for Science and Technology, Gamasa 35712, Egypt; 5 Pathology and Clinical Laboratory Medicine Administration (PCLMA), King Fahad Medical City, Riyadh 59046, Saudi Arabia; asealqahtani@kfmc.med.sa; 6 Department of Clinical Laboratory Sciences, College of Applied Medical Sciences, King Khalid University, Abha 61421, Saudi Arabia; moyahya@kku.edu.sa; 7 Department of Family and Community Medicine, College of Medicine, King Khalid University, Abha 61421, Saudi Arabia; aalsabaani@kku.edu.sa; 8 Institute of Tissue Regeneration Engineering, Department of Nanobiomedical Science, BK21 NBM Global Research Center for Regenerative Medicine, Dankook University, Cheonan 31116, Korea; rmurug2000@gmail.com; 9 School of Basic Medical Sciences, Chengdu University, Chengdu 610106, China

**Keywords:** nanoparticles, electronic nose, RT-PCR, biosensor, electro-chemical sensor, gas sensor, non-invasive sensing approaches

## Abstract

The COVID-19 pandemic has the tendency to affect various organizational paradigm alterations, which civilization hasyet to fully comprehend. Personal to professional, individual to corporate, and across most industries, the spectrum of transformations is vast. Economically, the globe has never been more intertwined, and it has never been subjected to such widespread disruption. While many people have felt and acknowledged the pandemic’s short-term repercussions, the resultant paradigm alterations will certainly have long-term consequences with an unknown range and severity. This review paper aims at acknowledging various approaches for the prevention, detection, and diagnosis of the SARS-CoV-2 virus using nanomaterials as a base material. A nanostructure is a material classification based on dimensionality, in proportion to the characteristic diameter and surface area. Nanoparticles, quantum dots, nanowires (NW), carbon nanotubes (CNT), thin films, and nanocomposites are some examples of various dimensions, each acting as a single unit, in terms of transport capacities. Top-down and bottom-up techniques are used to fabricate nanomaterials. The large surface-to-volume ratio of nanomaterials allows one to create extremely sensitive charge or field sensors (electrical sensors, chemical sensors, explosives detection, optical sensors, and gas sensing applications). Nanowires have potential applications in information and communication technologies, low-energy lightning, and medical sensors. Carbon nanotubes have the best environmental stability, electrical characteristics, and surface-to-volume ratio of any nanomaterial, making them ideal for bio-sensing applications. Traditional commercially available techniques have focused on clinical manifestations, as well as molecular and serological detection equipment that can identify the SARS-CoV-2 virus. Scientists are expressing a lot of interest in developing a portable and easy-to-use COVID-19 detection tool. Several unique methodologies and approaches are being investigated as feasible advanced systems capable of meeting the demands. This review article attempts to emphasize the pandemic’s aftereffects, utilising the notion of the bullwhip phenomenon’s short-term and long-term effects, and it specifies the use of nanomaterials and nanosensors for detection, prevention, diagnosis, and therapy in connection to the SARS-CoV-2.

## 1. Introduction

The entire globe is dealing with a devastating viral disease, COVID-19, which is caused by a corona virus, known as severe acute respiratory syndrome coronavirus-2 (SARS-CoV-2), that was first detected in November 2019 in Wuhan, China [1]. On 20 January 2020, the World Health Organization (WHO) designated the COVID-19 outbreak a worldwide public health emergency of international concern, affecting about half a billion humans (~0.45 billion), with more than 6 million deaths reported as of February 2022 [2]. After dealing with the initial blow (first wave) of COVID-19, several nations were confronted with more severe second and third waves of the pandemic. Over a span of more than two frenetic years, it was noted in [3] that there was no specific connection between the first wave, second wave, and third wave mortality rates, which rather depended upon the severity of the COVID-19 variants. History reveals that similar viruses have caused epidemics, namely the SARS-CoV in 2003 and Middle East respiratory sickness (MERS-CoV) in 2012. Despite being fractionally differing, in terms of genome sequencing, SARS-CoV-2 was found to be more devastating than its predecessors [4,5]. All these viruses are genetically categorized as ribo-nucleic acid (RNA) viruses, resulting in the transmission of infections to lymphocytes by incorporating RNA, which quickly replicates and collates viral proteins in host cells [5]. A SARS-CoV-2 infection should be validated by finding a unique RNA sequence, according to WHO standards. Reverse transcription polymerase chain reaction (RT-PCR) is a commonly used method, besides the antigen test (AT), for amplifying RNA sequences used for detection [6]. The AT was found to be less sensitive than the RT-PCR method, with an accuracy of about 63.5%, when compared to RT-PCR, which has an accuracy of about 94.7% [7] and mean test turnaround time of about 28.2 h [8]. Even with the use of the RT-PCR method, occurrences of false positive and false negative results are experienced. The need for an efficient, yet fast, method/approach that improves the accuracy of these tests was much needed. This thoughtful idea has given way to the use of nanotechnology, using nanomaterial-based sensors for effective testing.

**Figure 1:**
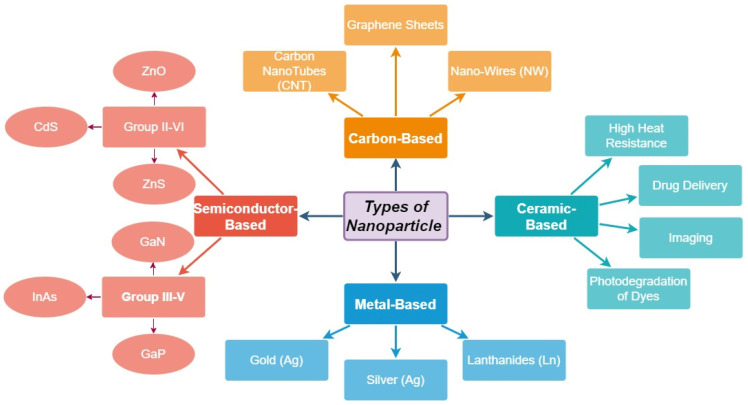
Classification of nanoparticles, in compliance with size, shape, and physical and chemical characteristics.

Nanomaterials are an arrangement of atoms and molecules that, when combined, create stable building blocks, which can further form a larger and more complex material or structure. Nanotechnology is the key to continuing improvement, in terms of performance and features, as technology advances. Nanoparticle-driven sensors have been proven to play a crucial role [9] in “Inactivating the pandemic” [10]. Proposed a method of capturing and inactivating SARS-CoV-2 using neutralizing antibodies attached to the surface of a photo-thermal nanoparticle (NP). Through surface neutralizing antibodies, the multifunctional NP effectively recognizes SARS-CoV-2 and completely prevents the viral infection of host cells, both in vivo and vitro environments. In addition to viral capture and blocking, the NP has a photo-thermal function, which generates heat after irradiation for virus inactivation. Nanoparticles are classified into various types, based on their size, shape, physical, and chemical characteristics. Carbon-based, ceramic, metal, and semiconductor nanoparticles are a few of the examples illustrated in Figure 1. Carbon nanotubes (CNTs) [11,12] and crystalline solids are the two major materials used in **carbon-based nanoparticles**. CNTs are just graphene sheets [13,14,15] folded into tubes. Because they are 100 times stronger than steel, these materials are primarily employed for structural reinforcement. CNTs are divided into two types: single-walled carbon nanotubes (SWCNTs) and multi-walled carbon nanotubes (MWCNTs). CNTs are one-of-a-kind in that they are thermally conductive along their length but non-conductive across their width. **Ceramic nanoparticles** are solids composed of oxides, carbides, carbonates, and phosphates. These nanoparticles are extremely heat resistant and chemically inert. They can be used in photocatalysis, dye photodegradation, medication delivery, and imaging. Ceramic nanoparticles may be used as an excellent drug delivery agent by manipulating several of their properties, such as size, surface area, permeability, surface-to-volume ratio, and so on; they are well-suited for nanomedicine. These nanoparticles have been successfully employed as a medication delivery method for a variety of disorders, such as bacterial infections, glaucoma, cancer, and others.

**Metal nanoparticles** are created chemically by reducing metal-ion precursors in solution using chemical reducing agents. They are used to create metal nanoparticles. Chemical, electrochemical, or photochemical processes can be used to create these nanoparticles. These have a high surface energy and the capacity to adsorb tiny molecules. These nanoparticles have uses in research, bio-molecule detection and imaging, and environmental and bio-analytical applications. For example, gold nanoparticles [16,17] are utilized to coat the sample prior to SEM analysis. Silver nanoparticles exhibits better antiviral action [18], which can be achieved through a single-step process using an extract of plantago major seeds [19]. Whereas a quick and precise transverse flow can identify SARS-Cov-2 in the blood samples of particular antibodies in serum [20]. **Semiconductor nanoparticles** exhibit characteristics that are similar to those of metals and nonmetals. They can be found in groups II–VI and III–V of the periodic table. These particles have broad band-gaps that change their characteristics, depending on how they are tuned. They are also employed in photocatalysis, electronics, photo-optics, and water splitting, among other things. GaN, GaP, InP, and InAs from groups III–V are examples of semiconductor nanoparticles. ZnO, ZnS, CdS, CdSe, and CdTe are II–VI semiconductors [21].

Nowadays non-invasive breath analysis approaches gained encouragement in the field of detection of SARS-CoV-2. The application of a revolutionary breath volatile organic compound (VOC) sensor technology [22] in a mechanical lung ventilation system is proposed by [23]. The sensor device in corporating metal oxide gas sensors and atmosphere sensors was mounted on the aeration exhaust port of the ventilation machine. This allowed for additional safety because the device is placed onto exterior of the contour between the patient and equipment. Signal registration was steady for many weeks, thus attributing to the effectiveness of the sensors. Using this electronic nose (breath analysis) approach, the sensitivity was found to be 90% in an illustration made by [24]. A successful presentation of an e-nose device with a sensor array, a data acquisition system, and machine learning that can identify changes of volatile organic compounds (VOC) present in the exhaled breath of patients and healthy beings with a 92.3% accuracy was produced [25]. For NO_2_ detection, classification, and discrimination among some of the most significant ambient toxicants, a miniature electronic nose (e-nose) based on a shear horizontal surface acoustic wave (SH-SAW) sensor array was presented by [26]. Acquiring real-time breath humidity data is critical for calibrating gas sensors and precisely monitoring a patient’s physiological information for non-invasive and point-of-care diagnostic applications. The successful humidity sensing characteristics of atomically thin 2-D metal oxide nano-sheets (NSs) produced by a liquid phase exfoliation technique were examined by [27]. A completely integrated OFET-based electronic nose was illustrated by [28], with the entire sensor module situated on a single substrate, capable of a detection limit of 30 parts per billion (ppb), as supplied by monolayer thick active layers that can function in atmospheric air with up to 95 percent relative humidity. Reference [29] put forward a technique that makes use of a newly designed breath device, which consists of a nanomaterial-based hybrid sensor module with multimodal detection capabilities that is capable of identifying disease-specific biomarkers in exhaled breath, thus allowing for quick and accurate diagnosis, with a sensitivity of about 90%.A detailed review of the methods, motivation, and ongoing challenges faced, with regard to the noise suppression, evaluation, and reliability was put forward by [30]. Colorimetric detection of coronavirus using nanoparticles is a simple, quick, and low-cost approach. Due to its coupling tendency with nanomaterials, gold nanoparticles (AuNPs)incorporated in electrochemical devices may even be useful in the detection of new kinds of coronaviruses in the future [31].

The National Aeronautics and Space Administration (NASA) replicated the human nose with the electronic nose(e-nose); it measures volatile organic compounds (VOC), which cannot be achieved by biological noses [32]. The VOCs contains the gases generated by an infection from a virus, such SARS-CoV-2, which causes the COVID-19 illness. Electronic olfaction employs data processing and machine learning to create prediction model based on the feedback of multiple sensors, in the form of multivariate datasets, thus allowing it to distinguish between illness and healthy controls, based on a unique imprint [33]. A testing accuracy of about 93% was achieved in an illustration provided by [34], using principal component analysis (PCA), random forest, and linear discriminant analysis (LDA). It was illustrated clearly by [35] that, by incorporating machine learning approaches such as PCA and canonical analysis, sensitivity reached its full-scale value of 100%. This non-invasive platform was carried out successfully by [36], for integration into protective masks to capture airborne virus in exhaled breath throughout the course of utilization. To simulate airborne dispersion, a viral sample was injected into the collector, and the enhanced pathogen was then recovered for additional analytical assessment. Nano ecosystems can improve the specificity/efficiency of immunosuppressive drug delivery to target immune cells, resulting in lower drug doses, less drug distribution to non-target tissues and organs, and fewer possible side effects [37].

**Figure 2:**
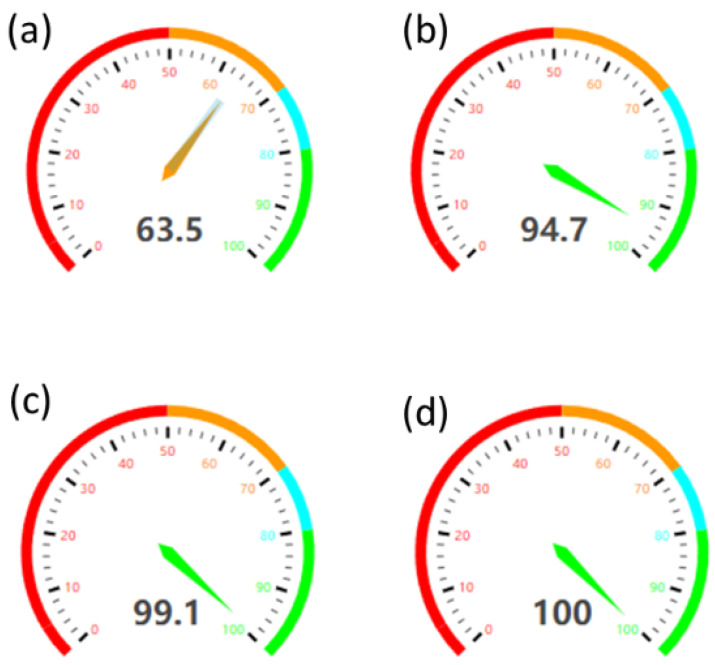
Testing procedures accuracy. (**a**) Antigen test (AT), (**b**) RT-PCR, (**c**) nanomaterial embodiment testing, and (**d**) amplification and multiplexing of genome with nanomaterial FET embodiment testing.

This comprehensive review article puts emphasis on the most recent developments in nanotechnology-based sensors, focusing on the synergy between the virus detection (offered by the nano-sensing mechanism and assay techniques) [20,38,39,40,41,42,43,44,45], diagnosis (using nanostructured drugs and nanomedicine) [15,46,47,48], and prevention (design of face masks, gloves, and personal protective equipment) [49], thus offering a holistic survey, concerning their usability and performance, in contrast to previous review papers [18,50,51,52,53,54,55,56,57,58,59], which only discussed parts of them. The embedment of nanotechnology for the prevention, diagnosis, and detection, in order to battle novel COVID-19, concerning the point of care (POC), was briefly discussed and concluded in [11,60,61,62,63]. For instance, an electro-chemical nanomaterial-based sensor was put forward, showing an improvement in testing accuracy of about 99.1% [64]. Additionally, it was illustrated by [65] that, by amplification and multiplexing of the SARS-CoV-2 genome with the nanoparticles embedded in a field-effect transistor (FET) device, the achieved detection accuracy was found to be 100%. A comparison of accuracies for various testing approaches is illustrated in Figure 2.

Deltamutants within the SARS-CoV-2 (COVID-19) virus continued to cause havoc, with high infection rates and mortality, highlighting the lack of efficacy of the SARS-CoV-2 vaccine. However, with the use of graphene quantum dots bound to human host defense peptides, it has been reported that it helps to prevent the virus from invading host cells [66,67]. Human cells are often infected with the coronavirus in congested areas, as the virus turns its host into a viral replicator. Scientists at Stanford University used a high-resolution light microscope, for the first time, to classify and locate the virus molecules inside the cells [68,69].

**Scheme 1:**
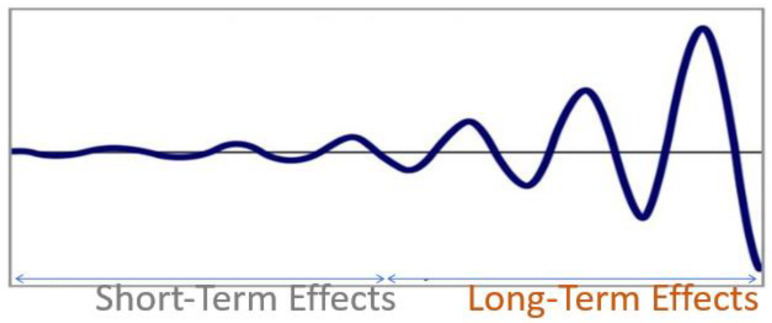
Bullwhip phenomenon [70,71].

Since the on-set of the pandemic, the main concern of exploration put forward by the researchers revolves around the detection, diagnosis, and prevention approaches. A keen study is yet to take place, concerning the circumstances experienced due to the after effects of the pandemic. This review article, in addition to the detection, diagnosis, and prevention of COVID-19, also discusses the consequences yet to be experienced by society, using a popular approach known as the bullwhip phenomenon. The bullwhip phenomenon is an event that occurs in the operations department. It claims that any tiny disruptions in the supply chain can cause extreme changes in supply and demand satisfaction. This effect is named after the physics associated with cracking awhip. When a person with a whip snaps his wrist, the pattern of the whip wave gradually strengthens in a chain reaction of relatively small movements. If the consumer supply chain is related to the “short-term effects” that are currently being experienced globally, the changes in supply and demand satisfaction will point towards “long-term effects”, which are expected to be catastrophic and yet to be experienced by society as a whole [70]. Scheme 1 illustrates the short- and long-term effects of those effects, both during and after the pandemic.

The short-term effects that are currently being experienced by the society include degradation/unlikeliness in ability to return to work, employee furloughs, employees work and non-work interface imploded, financial loss experienced by travel, entertainment, and restaurant entities, increase in temporary/contracting/third-party employment, and rapid transition to remote work, thus resulting in system crashes or inconsistent access, along with some social and psychological impairments. The long-term effects are yet to be experienced by society as a whole, which includes individuals being financially prepared to spend less, in order to be ready for upcoming shortcomings.When organizations fail to meet customers’satisfaction demands, it results in greater debts. Job designations that can transform easily to remote work will gain increased willingness and preference; return to normal will increase stress among employee with kids, as they gained peace during the work from home period.

The following are some of the resultants that give rise to the bullwhip phenomenon.The next best decision is made by supply chain stakeholders at each point in the chain or customer service or delivery. It reacts due to a lack of communication and coordination between each link in the supply chain or stakeholder, when the expectations of demand are excessively estimated or underestimated. Too many or insufficient orders andlead-time issues causeproduction delays. Customers, often retailers, wait for orders to accumulate before placing an order with a supplier. This is a method called “order bundling”. Discounts, cost changes, and other price fluctuations disrupt normal buying behavior. Inaccurate forecasts, due to excessive reliance on past demands to forecast future demands, and lead-time issues causeproduction delays.

## 2. Fabrication of Nanomaterial Structures

The prefix nano comes from the ancient Greek word “nanos”, which signifies “dwarf” or “small”. Nano is a prefix that denotes a billionth (10–9) of a quantity. At this particular level, the molecules or atoms are arranged so that, when combined, they create stable building blocks that evolve into more complex, larger materials and structures. These structures are refereed as nanomaterials. In a nanomaterial, the surface/boundary/interface plays an important role. The surface area-to-volume ratio tends to rise when the material’s fundamental length dimensions decrease and vice versa. As the size of the material shrinks, a greater proportion of the atoms are located on the surface than in the bulk or inside the same substance. A given mass of nanomaterials will be far more reactive than a similar quantity of material made up of bigger particles when growth and catalytic reactions occur on the surface. A nanostructure is a term that includes materials having a size of 1–100 nanometer (nm) in various dimensions (D), namely 0-D, 1-D, 2-D, and 3-D.

All dimensions in zero-dimension (0-D) nanomaterials are measured on the nanoscale, which implies no dimension is bigger than 100nm. Nanoparticles and quantum dots are best examples of 0-D nanomaterials. In one-dimensional (1-D) nanomaterials, all dimensions are measured on the nanoscale, with the exception of one dimension. Examples of this class include nanowires, quantum wires, nanorods, and nanotubes [72]. The one dimension confined out of nanoscale is generally used for electrical conduction; this feature allows for its use in electrical induction applications. Nanowire is a structure with a thickness or diameter of about 10nm. It can be configured to be conducting, semiconducting, or insulating. Due to its unique capability of electron density, it exhibits significant optical, electrical, and magnetic properties. Carbon nanotubes are elongated tube-like structures with walls made of thick carbon or graphene sheets. The attributes of nanotubes are altered by rolling these sheets at precise chiral angles and a quantized radius. Individual carbon nanotubes are held together by van der Walls forces. They exhibit extra ordinary thermal conductivity and mechanical and electrical properties. An inorganic nanotube is made out of metal oxides and has a number of benefits, including being synthetic, crystalline, homogeneous, adhesion to a variety of polymers, and excellent impact resistance. Boron nitride and silicon carbon nanotubes are two examples of inorganic nanotubes that are both resistant to oxidation and suitable for high temperatures [73].

**Figure 3:**
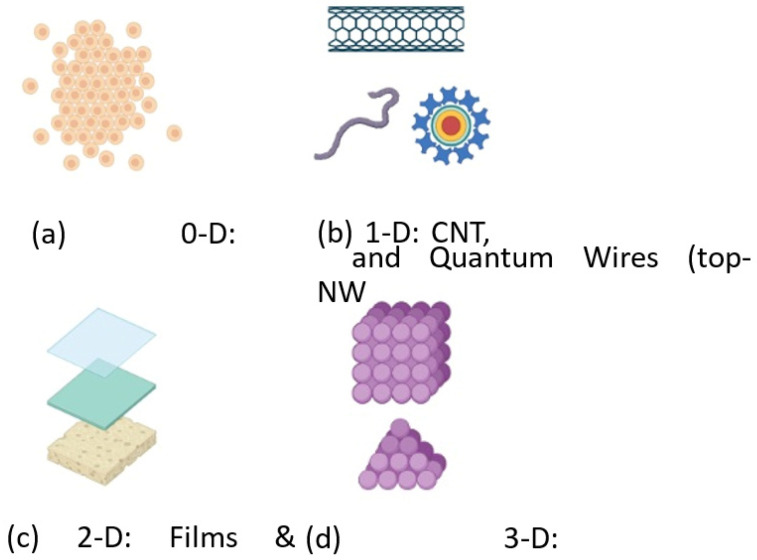
Nanostructures;(**a**) 0-D, (**b**) 1-D, (**c**) 2-D, and (**d**) 3-D.

Two-dimensional (2-D) nanomaterials have all of their dimensions measured inside the nanoscale, with the exception of two. Thin films, planar quantum wells, and super-lattice structures are notable examples of this class of dimension. The two dimensions out of the nanoscale comprise electrons and photons, which affects the wave function, density of states, and thermal transport, respectively [74].The three-dimension (3-D) nanomaterials are the ones in which all the dimensions are confined out of nanoscale. Bulk nanocrystalline films and nanocomposites are examples of this class of structure. The general illustration of 0-D, 1-D, 2-D, and 3-D is illustrated in Figure 3.

Fabrication at the nano-scale, or simply nanofabrication, involves the synthesis of nanomaterials or structures, in order to alter the properties of naturally available materials, which results in improved the energy efficiency, sustainability, and standards of the society we live in. Nanofabrication is divided into two types of approaches: top-down and bottom-up.

### 2.1. Top down Approach

A top-down approach is one which seeks to build by starting with a larger component and carving away the material to change its properties. Specifically, in nanofabrication, patterning and etching away material signifies this type of approach. It employs nanofabrication techniques that rely on external instruments to regulate the cut, mill, and shape. Photolithography and inkjet printing are examples of micro-patterning processes. In this section, techniques such as focused ion beam (FIB), photolithography, e-beam lithography, and magnetic particle spectroscopy are discussed in detail.

For the deposition and ablation of materials, **focused ion beam (FIB)** is a technology utilized mostly in the semiconductor industry, material science, and biological sciences. As the name illustrates, FIB systems employ a directed ion beam, rather than an electron beam; in particular, the beam of ions is made up of gallium. **Photolithography**, also known as optical or ultraviolet (UV) lithography, is a technique for crimping portions of a specimen in nanofabrication. As the name signifies, it uses a photon beam to crimp the desired pattern using a light-sensitive chemical photoresist layer [75]. **Electron beam (e-beam) lithography** is a method that employs a beam of negatively charged particles, in order to produce the circuit patterns required for the material deposition on the wafer or extraction from the wafer. E-beam lithography possesses shorter wavelengths of about 0.001–0.005 nm, thus offering a higher performance resolution throughout the process. Patterns with a low resolution of roughly 10 nm may be produced using electron beam lithography. This technology allows for the scanning of an electron beam over a surface, thus eliminating the need for masking and accomplishing the removal of atoms by simply setting boundary lines, as in the case of a drawing pen. **Magnetic particle spectroscopy (MPS)**, often referred as magnetization response spectroscopy, is a flexible, highly sensitive, low-cost analytical detection method for a wide range of biological and biomedical investigations. It is based on magnetic particle imaging (MPI), which measures the density of super magnetic iron oxide nanoparticles directly. As a result, MPS may be seen as a zero-dimensional MPI scanner capable of conducting spectroscopic investigations on hyper magnetic iron oxide nanoparticles [76,77]. Spectrometry is often used for the detection of the volatile organic compounds (VOCs) found in human breath [78].

### 2.2. Bottom up Approach

In contrast to the top-down approach, which involves removing the materials that are not required, the bottom-up approaches rely on fundamentally building pieces to provide the required characteristics. This is accomplished by self-assembly, which eliminates the requirement for patterning. **Self-assembly** is described as the spontaneously and reversible arrangement of molecule components into ordered structures at the atomic or subatomic levels by non-covalent contact forces. In simple words, self-assembly refers to a nanostructure that builds itself. Bottom-up techniques may produce devices concurrently, and they are substantially less expensive than their top-down counterparts. As the scale and complexity of the required assembly grows, this strategy may become too much to handle.

**Figure 4:**
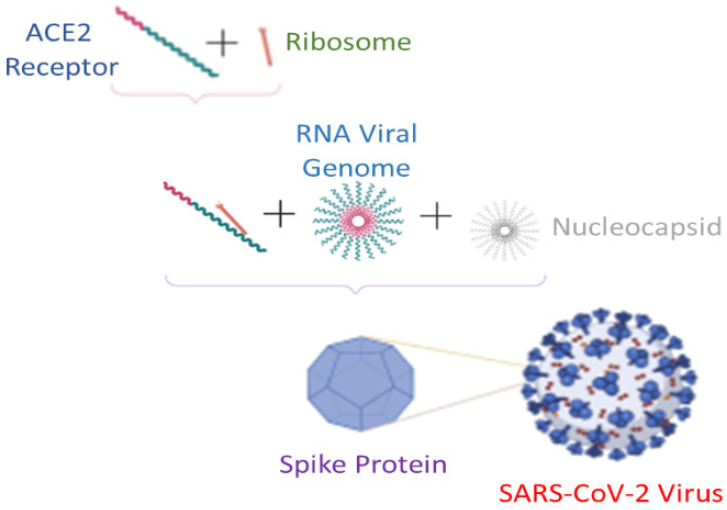
Self-assembly in spike protein formation.

Intra-molecular and inter-molecular self-assembly are the two forms of self-assembly. Intra-molecular self-assembly molecules are frequently complex polymers, capable of assembling from a random coil conformation into a well-defined stable shape. Protein folding is an example of intra-molecular self-assembly. Inter-molecular self-assembly, on the other hand, is the capacity of the molecules in a sample to create molecular assemblies. One of the inter-molecular instances is the creation of mycelia by-molecules in solution, which is a basic example. In nature, self-assembly happens spontaneously. Self-assembly is used by many biological systems to bring together different molecules or structures, as illustrated in Figure 4.

Consider a virus as a tree that is developing and branching out; each arm is somewhat different from the others. Experts in the field can categorize the branches, based on their distinctions, by comparing them. Since the start of the pandemic, these little changes, or variants, have been examined and recognized. As the virus spreads, it will have more possibilities to evolve, making it more difficult to eradicate. These changes can be tracked by comparing physical features (such as treatment resistance) or genetic code changes (mutations) from one version to the next [79]. In other words, it can be thought that these variants occur based on the self-assembly phenomenon, experiencing changes with the virus genome recombination with the host cell [80,81,82,83,84,85,86,87]. The variants of concern are a few of the many named using Greek letters, such as ALPHA, BETA, GAMMA, DELTA, EPSILON, ZETA, ETA, THETA, IOTA, and KAPPA, in order to avoid stigmatizing the nations where they were first detected.

## 3. Synergy between COVID-19 Prevention, Detection and Diagnosis Using Nanosensors

**Figure 5:**
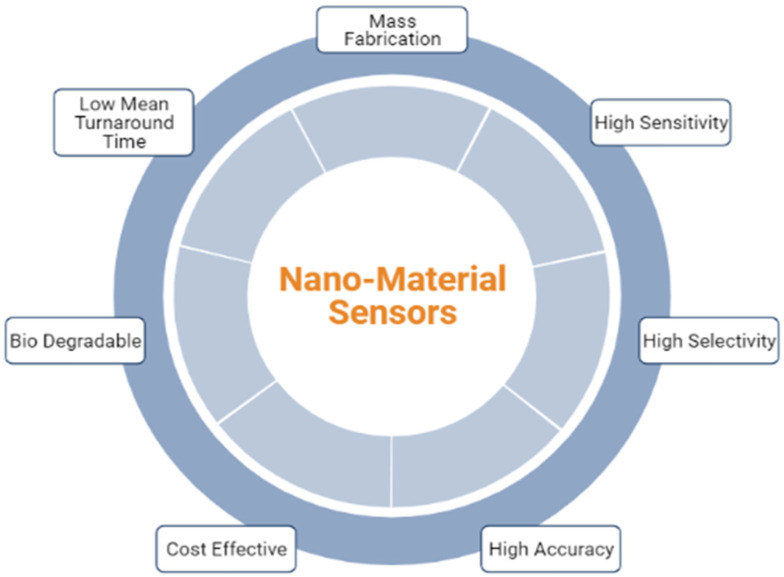
Salient features of nanomaterial-based sensors.

Tedious sample processing techniques evolved in traditional laboratory-based diagnostic examinations. Wearable [88,89] and portable [47] biosensors have gradually gained considerable attention as a way to encourage non-invasive measurements and continuous monitoring, thereby achieving greater efficiency. Wearable sensors are effective for addressing mass-level screening and providing point-of-care “*prevention*”, which is critical in preventing disease spread [90]. With a nanomaterial as the base material, cross-relative or simply hybrid sensors also plays an important role in increasing the sensitivity and accuracy of the traditional approaches [91,92]. Figure 5 shows the salient features of a nanomaterial sensor.

COVID-19 detection using nanomaterial-based sensing through surgical masks is identified using a hydrophobic polypropylene (PP) layer, which offers liquid barrier protection in commercial surgical masks [93]. This layer captures the aerosols and droplets created while the wearer breathes and talks during the use of a surgical mask. It is expected that SARS-CoV-2 antigens would collect in this layer, as well, where they might be detected using nanoparticle transfer biosensors. These biosensors are made of filter paper that has been polymer-modified, in order to hold using antibody-decorated gold nanoparticles. The polymer prevents irreversible interactions in between the paper and nanoparticles in dry circumstances, thus ensuring complete nanoparticle release when liquid is applied [94]. The World Health Organization (WHO) advises using physical and chemical variables to reduce contamination, such as masks and personal hygiene routines, as well as disinfecting surfaces, particularly those that are regularly handled, such as door handles, tables, chairs, handrails, and switches. The use of personal protective equipment (PPE), disinfectants, and sanitizers is critical in this situation for the appropriate containment of the disease [95]. Researchers at the Massachusetts Institute of Technology (MIT) employed a freeze-dried cellular machinery technique to develop nanosensors for use in paper diagnostics embedded in a face mask for viruses, such as Ebola and Zika [96,97].

**Figure 6:**
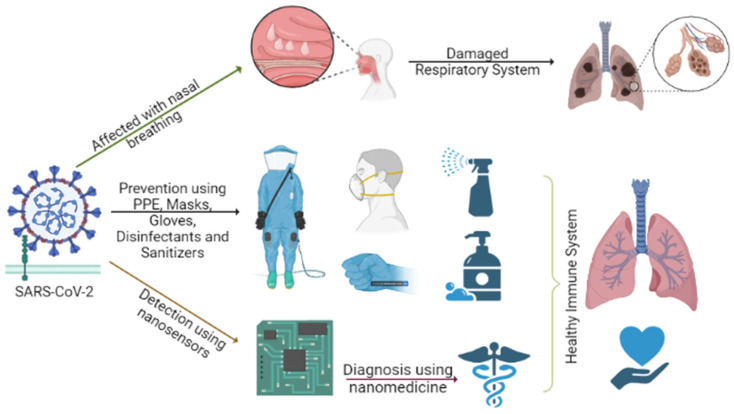
Prevention, detection, and diagnosis of SARS-CoV-2.

Viruses are miniature biological structures with a diameter of a few micrometers (in the case of SARS-CoV-2, the size ranges from 0.1µm in diameter). The tests for COVID-19 “*detection and diagnosis*”, in particular, are based on specific nucleic acids and proteins, as well as point-of-care testing and use of biomedicine and nanomedicine [98,99] for post-infected patients, as well as vaccinations for both pre-infected and post-infected patients [100]. Nanotechnology, with its applicability, is an efficient and cost-effective method for improving these detection tests. As discussed in the earlier section, a larger surface-to-volume ratio of nanomaterials finds its way in for sensing applications. Reference [101] discussed an efficient way of using a colorimetric-based approach, incorporating gold nanoparticles for the detection of viruses, along with anti-viral nanomedicine. The authors in [102] put forward the discussion of bridging the gap between the laboratory testing, opted for during pre-pandemic and clinical applications, to be prioritize during and after the pandemic span [103]. Figure 6 illustrates the prevention, detection, and diagnosis implication of using nanomaterials. Owing to their drug loading/releasing capacities and possible photodynamic/photothermal therapeutic qualities, nanomaterials with enhanced functionality are especially intriguing [104].

**Figure 7:**
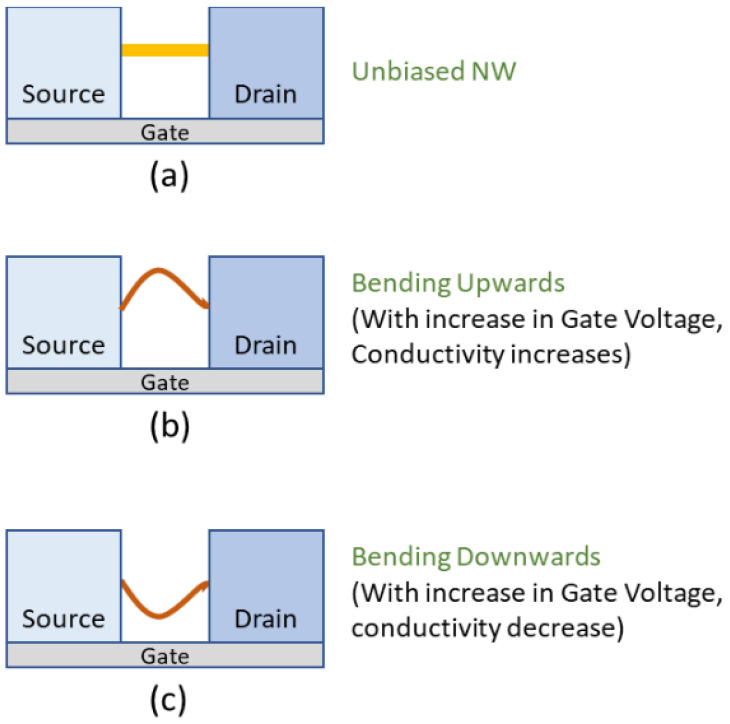
(**a**) Silicon (Si) nanowire (NW), (**b**) p-type Si NW, and (**c**) n-type Si NW.

A nanowire (NW) can simply be termed as a material in a thread or strip formed at the nano-scale. As a 1-D class of a nanomaterial, it exhibits a large surface-to-volume ratio, thus empowering its use for detecting charged particles, as well as its use for field sensors. NWs are employed in situations where electron transfer, rather than tunneling mobility, is required, due toto their superior and distinctive electrical conveyance characteristics. A typical Si NW, along with its p-and n-type differences, are shown in Figure 7a–c, respectively. A p- or n-type nanowire with metal electrons at both ends is contacting between two electrodes and integrated in a platform of field-effect transistors, in order to well-understand the concepts of NWs. The silicon nanowire bands bend ‘up’ for p-type silicon nanowires and ‘down’ for n-type silicon nanowires, similar to a standard metal semiconductor interface. The bands are lowered when a positive voltage is applied to NW. As a result of this activity, the holes in p-type silicon nanowires are depleted, and the conductivity of the silicon nanowires is suppressed. The weakening of the bending bands in n-type silicon nanowires causes an influx of electrons in the n-type silicon NW, which improves conductivity. Whenever the gate voltage is raised, the conductivity of p-type silicon NW increases, while the conductivity of n-type silicon NW decreases. It may be deduced that a nanowire’s operational bands occurs in the conduction band. A carbon nanotube (CNT) is a tube-shaped material made of carbon (in particular, graphene), forming a closed cylinder structure with a diameter that is measured at the nanometer scale. When compared to other materials that normally possess one or more of these features, CNT has a unique mix of rigidity, strength, intensity, thermal, and electrical conductivity.

In this section, the sensing applications are discussed for 1-D structures, such as nanowires (NW) and carbon nanotubes (CNT). Various sensors, such as chemical, gas, DNA, electrochemical, and optical sensors, are discussed in this section. During the pre-pandemic era, the utilization of nanomaterials for detection and diagnosis was neglected, due to the availability of procedures for various diseases. During the pandemic, nanomaterial-based sensors gained popularity, due to their high sensitivity, accuracy, and the fact of the unavailable resources for completely defending against the spread of virus. Researchers around the globe looked for similar ways and scenarios in which nanomaterials are used as sensor for detecting pathogenic viruses [105,106], along with the four commonly damaging viruses, namely influenza, hepatitis, HIV, and dengue [107].

### 3.1. Chemical Sensor

**Figure 8:**
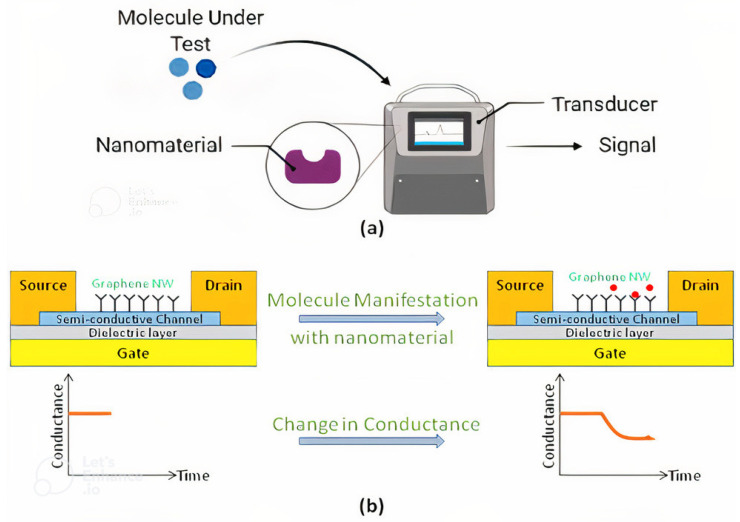
Chemical sensor (**a**) setup and (**b**) mechanisms.

Chemical sensors convert chemical data, such as the proportion of a given sample component, into an analytically usable signal. Chemical information is converted into energy that can be detected by a transducer in the receptor component of a sensor, which is clearly illustrated in Figure 8a. Utilizing a field-effect transistor (FET) device, the sensing method can be customized (CNT-, NW-, or graphene-based) [48,108,109,110,111], as illustrated in Figure 8b. The customization of the FET device, accomplished using quantum dots, is illustrated by [101]. The target molecule is normally manifested in aqueous solution and has a net positive or negative charge, due to binding. The identification of the target molecule can be accomplished by measuring the conductivity of the graphene-based nanowire device [14,55,112,113,114,115]. Hence, a silicon NW enables multiplexed fast detection, with great selectivity and sensitivity. A chemical sensor made with CNT helps in detecting traces of explosives and toxins in water. The capacity of a CNT to detect trace amounts of explosive and chemical hazardous agents in an aqueous chemical sensor has been demonstrated by [116]. The analytic response of the single-wall carbon and cube-based thin film transistor device is highly dependent on the sensor’s network structure. Close to the threshold, a network of unbundled mainly semiconducting nanotubes gives high sensitivity and reversibility. When subjected to substantial bending deformations, this type of nanosensor maintains its transconductance, carrier mobility, and on/off ratio [117,118].

The capacity to sense and respond to one or more stimuli reversibly is one of the characteristics of smart materials. When the stimuli are withdrawn, the animals revert to their previous condition. These stimuli might be external or internal to the sensor device, which can be found in chemical sessors, gas sensors, etc. [119]. Several studies have shown that using CNTs, either functionalized or in conjunction with other materials, can help improve their characteristics, which enables the design of simple, yet extremely sensitive, mass-produced biosensors for its use, which not limited to the COVID-19 pandemic, but also future pandemics [28,120]. A two-step solvo-thermal approach was used by the researcher in [121] to manufacture a new nanocomposite, based on novel spinel copper cobaltate(CuCo_2_O_4_)nano-sheets and N-doped graphene quantum dots (N-GQDs) as an iron-free heterogeneous Fenton-like catalyst.

### 3.2. Gas Sensor

The use of silicon nanowires as gas sensors is fraught with difficulties, as the silicon oxide and interface have a high density, regarding the surface states. When employed as a sensor, the presence of native silicon oxide on the silicon (Si) nanowire surface reduces the sensitivity of the field-effect transistor device [122]. Furthermore, at the air/silicone oxide interface, silicone nanowires employed, or used as gas sensors, suffer from high density, regarding the trap states [123]. The hysteresis phenomenon, in the response obtained in the forward and backward electrical scans of the source drain current versus back-gate voltage, is caused by the process of the native silicon oxide surface, which is liable for the surface substrates that induce the hysteresis tendency or merely a slowdown in the determinant [124]. The use of a monolayer molecule to functionalize the silicon nanowires oxide sheath to decrease linkages is an efficient way to eliminate trap states and the hysteresis effect.

The examination of a gas sensing application, assessed at room temperature using FIB equipment, revealed that hydrogen gas has a high responsiveness and selectivity, when compared to widely utilized gases, such as carbon dioxide, carbon monoxide, ammonia, etc. [125]. As illustrated by [126], in order to enable silicone nanowires to sense volatile organic compounds or gas anolytes, the silicone nanowire shall be modified with a dense organic monolayer. At high negative back-gate voltage, the measured current between the drain and source is nearly equal to the current seen between drain and source in the air. When positive gate voltages are applied, the drain-source current decreases systemically, thus lowering the concentration of volatile organic molecules. The silicon oxide monolayer interfaces are modified as a result of the effective dipole moment of the organic monolayer state density, which alters the conductivity of Si NW [122].

CNTs for gas sensing applications use processes that are similar to those found in liquids. They interact non-selectively with a wide range of gases, often with high binding energies, thus resulting in extended time constants for gas deposition [127]. As a result, carbon nanotube sensitivity, selectivity, and reversibility are improved by generating flaws along the sidewalls of the carbon nanotubes using various chemical and physical treatments. Acid oxidation, for example, increases the surface area of carbon nanotubes, develops sidewall flaws, and lowers functional groups at the defects on the nanotube surface, all at the same time. Carbon nanotube oxidation improves its reaction to polar vapors, including water vapor, in addition to organic and inorganic gas responses, thus posing a serious challenge regarding water interference [128]. The electrical characteristics of the semiconductor carbon nanotube surface are altered by charge transfer, which is generated by the absorption of polar organic molecules.

### 3.3. DNA Sensor

Chemical vapor deposition methods are used to build random networks of single-walled carbon nanotubes, with diameters ranging from 1–3 nm and lengths ranging from 5–10 µm, on silicon oxide wafers [129]. The single-walled carbon nanotube network transistors are fabricated in top contact device geometry. To eliminate the weakly attached DNA molecules, conventional washing is used. It is obvious from this that the attachment of DNA molecules to the side walls of single-wall carbon nanotubes is responsible for the significant drop in drain current. Furthermore, in many recent studies [130,131,132,133], threshold voltage swing is considered a bio-sensing metric [134], rather than using the drain current of the device, thereby resulting in electron doping of single-wall carbon nanotube semiconducting channels.

### 3.4. Electro-Chemical Sensor

Researchers demonstrated that a cotton-based CNT exhibits high sensitivity and selectivity for detecting albumin, a protein of importance available in blood samples [135,136]. Typically, a cotton strand varies from 10–65 µm in diameter. In this approach, cotton strand/paper sheet is coated with a combination of CNT and poly-cations or poly-anoins, which are electrolytic in nature, with high molecular weight. The obtained by-product exhibits high electrical conductivity [137]. When the carbon nanotube cotton/paper cloth incorporated anti-albumin, it can be thought of as an electronic textile biosensor [138,139]. Single- and multi-walled CNTs are dispersed in dilute ethanol or water solution. Next, the cotton thread is dipped in the prepared carbon nanotube dispersions and dried. It is to be noted that the desired level of resistance required will not be achieved by single dispersion; repetitive dispersions or strewing are prolonged to accomplish a resistivity of about 20Ω/cm, which is minimally required to power a light emitting diode (LED) connected to a battery constructed using cotton-based CNT threads. The mechanical strength of the by-product is observed to be doubled, when compared to the original cotton strand, but still retains its flexibility suitable for its use in wearable electronics applications and helps in fighting the cytokine storm [140,141,142,143,144].

### 3.5. Optical Sensor

When single-walled CNTs are put to a dye-labeled, single-stranded DNA solution, a hybrid structure is created, wherein the di-molecule is in close vicinity to the nanotube, thereby suppressing the dye molecule’s fluorescence. In the presence of the target, dye-tagged, single-stranded DNA can reinstate the fluorescence signal to its initial condition. Furthermore, the sensitivity (S), which is defined as the change in resonant shift over a unit change in refractive index, is the most often utilized performance parameter in the case of such optical sensors [14,145].

## 4. Conclusions

The COVID-19 pandemic highlighted the crucial need for clinical diagnostics, detection, and prevention to be redesigned, in order to implement new technologies for point-of-care (POC) testing with enhanced precision and consistency. People are adjusting to the pandemic’s new organizational needs, while hoping for a quick “return to normal”. The ramifications of these demands may not only alter course throughout time, but also have a long-term impact on how businesses and workers operate, in short, adapting to the “new normal”. As per this review, using the investigations upon the utilities of nanosensors, it is identified that nanobiosensing devices have made significant advances in infection recognition throughout aspects of selectivity, affectability, and reaction time. A detailed discussion regarding the synergy between COVID-19 detection, diagnosis, and prevention was met using chemical sensors, gas sensors, electrochemical sensors, DNA sensors, and detection with FETs, carbon nanotube, and nanowire-based biosensors, as well as optical sensors and smart and wearable biosensors. The NW-based FET sensor was found to be the best choice for detecting micro- and nano-scale analytes. Although such devices have several advantages, one drawback is the presence of background electrostatic noise, which might restrict the sensor’s efficiency. Recognizing the centrality of biosensors in the diagnosis and management of infections, it is important to overcome the existing hurdles related to their sensitivity, specificity, mobility for point-of-care devices, and commercialization cost. Current biosensor research attempts to express an insight of robust biosensors capable of regeneration for reuse, thus allowing for long-term usage and the reduction of cost concerns. The successful integration of polymers, nanomaterials, and biology appears to be the key to producing better and more potent biosensors. Furthermore, due to the sheer adaptability of cutting-edge procedures, their frameworks might be improved to obtain greater outcomes at a quicker rate. As mentioned in earlier sections, there has been an increase in the use of nanomaterials for sensing mechanisms. Various future investigations on this subject might be carried out, taking advantage of the vast value of these methodologies. Multiple layers for the detection and freezing of viruses/bacteria discovered in breath might increase detection quality. Nonetheless, the technology might be used with a model to determine whether sufficient physical space is maintained between individuals. It might also be combined with a design that recognizes the kind of mask, mask functioning, or time to replace a person’s mask.

## Data Availability

Not applicable.
